# Supplementary material for: Facebook as an engagement tool: How are public benefit organizations building relationships with their public?
Source: PLoS One. 2021 Sep 9;16(9):e0256880. doi: 10.1371/journal.pone.0256880 (PMC8428678; doi:10.1371/journal.pone.0256880)
Supplement: Attachment — Submitted filename: Response to Reviews.docx [file pone.0256880.s001.docx]

**Answers to Editor**

1. Manuscript meets PLOS ONE’s style requirements (according to The PLOS ONE style template)

2. We have changed the title of the manuscript as suggested*: Facebook as an engagement tool: how are public benefit organizations building relationships with their public?*

*3.* All relevant data are within the manuscript.

We want to change the Data Availability statement. There is no repository information for our data at acceptance (it is explained in the cover letter).

4. Financial statements

a) The research did not receive any financial or material support.

b) We state: *The funders had no role in the study design, data collection and analysis, decision to publish, or preparation of the manuscript.*

c) The authors did not receive any salary.

d) The authors received no specific funding for this work.

**Answers to reviewers**

**Reviewer 1**

1. The structure of the theoretical part has been improved. The literature was associated with the research part of the work - especially with hypotheses.
2. Some results described in the paper have a universal character and they may apply regardless of the country. Legal solutions used in Poland force organizations with PBO status to communicate with an individual to encourage them to donate funds to the organization. Despite the lack of such solutions in other countries, the behavior of these organizations is similar, as they also have to communicate with people from their environment in order to raise funds.
3. This article is part of a larger research project that analyzed the use of social media in the activities of non-profit organizations. The conclusions from the use of Twitter and Facebook clearly differ. Only similar research procedures were used, while the conclusions were different. Twitter builds engagement with the public in a different way than Facebook, and its role in Polish organizations is much smaller (it does not have the same application as in the case of Anglo-Saxon countries).
4. The research sample was selected in a traditional manner and in accordance with the rules of statistics (stratified sampling). The methods of data collection using artificial intelligence do not provide the possibility of full analysis. The manual analysis made it possible to select the organizations that will be the case studies in subsequent publications. Manual research allowed to identify all the nuances of using Facebook in the surveyed organizations. In addition, obtaining financial data about the organization was not possible with the use of artificial intelligence.
5. Corrected.
6. The comment was taken into account in full. The literature part has been shortened. Only relevant fragments were left (ideas and findings were presented concisely). The text was checked once more by the company providing services to the University of Warmia and Mazury in Olsztyn (attached confirmation) and submitted to a native speaker (Michael Thoene) for checking.

**Reviewer 2**

1. Corrected (specific information on methods added).
2. According to the list of points (a-f)

2a. corrected - only the second part of the hypothesis remained,

2b. corrected - hypothesis verification is placed under each section,

2c. corrected - some results that did not test any hypotheses were removed (The fragment 528-560 was deleted). We left fragment 498-527 (the lack of communication tools in posts would make it impossible to confirm or reject hypothesis 2),

2d. corrected in accordance with the reviewer's comment,

2e. corrected,

2f. corrected (H4 was split into two hypotheses H4 and H5).

3. Improved throughout the article

**Reviewer 3**

1. This article brings the following contribution to the scholarship literature. To begin, it builds on the effectiveness of computer-mediated organizational communication. This is the first organizational-level study of Facebook effectiveness with a unique type of nonprofit organization with the ability to receive 1 % of personal income tax. The Polish case may be a valuable example for the implementation of similar solutions for nonprofit organizations in other countries. The growing importance of social media in the activities of nonprofit organizations requires taking steps to create effective strategies for their use. This is especially important concerning limited resources that usually nonprofit organizations own. We consider "online engagement" as a vital intermediate objective, which should result in real world activities by the organizations’ online audience (e.g. do‐ nation, voluntary work, lobbying).Therefore, it can be concluded that despite the increasing technological advancement and the use of sophisticated information exchange techniques, the organization itself and the ideas it presents are still the most important (this can later be translated into content published in online channels). It is important, therefore, that a given idea and related contents arouse controversy, evoke strong emotions, are up to date, etc. As an example, you can give an action on Facebook "Stop lawlessness" regarding the reform of the Polish judiciary and related EU objections regarding the state of law in Poland. This problem caused much controversy in Poland and "the real world" protests were current, that's why posts associated with this event attracted a large crowd of followers, and consequently triggered a large audience reaction. Another example are the posts about extreme animal suffering, which can also trigger a similar reaction. The use of sophisticated communication tools in this case, such as a hashtag, mention or hyperlinks, was not very important and only fulfilled an auxiliary role. Thus, it can be seen that social media, despite different ways of disseminating information (compared to traditional media), are still grounded in real problems, and the number of followers (observers) does not depend on technological advancement in the way information is provided (e.g. post construction), only from the "real" problem. This principle seems to be universal, i.e. it applies regardless of the country, because human behaviour in this respect is rather similar.
2. Interactions were checked. The entire research was done by hand, so with a high degree of probability it can be said that they were not bots. We have not studied the nature of the interactions (whether they are positive or negative). Audience analysis will be the subject of separate studies.
3. The research sample was selected in a traditional way and in accordance with the rules of statistics (stratified sampling). Therefore, not all the organizations from group 1 were examined, despite their small number (64 entities out of 77).
4. Page 15 – line 355-359, corrected.
5. Eliminated typos. Forwarded to proofreading done by a native speaker.
