# Supplementary material for: Facebook as an engagement tool: How are public benefit organizations building relationships with their public?
Source: PLoS One. 2021 Sep 9;16(9):e0256880. doi: 10.1371/journal.pone.0256880 (PMC8428678; doi:10.1371/journal.pone.0256880)
Supplement: Attachment — Submitted filename: Answer to reviews.docx [file pone.0256880.s002.docx]

**Answers to reviewers**

1. **We have changed the subheadings and text within them to avoid repetitions from other papers.**
2. **We have changed the hypothesis 2 (H2).**
3. **Novelty and originality of the research was underlined by adding, the following sentences to the introduction part of the article (some sentences were also moved or deleted):**

*Research conducted on the organizational uses of social media has primarily been concerned with commercial, for-profit entities. The scholarship literature on non-profit organizations is much sparser. This is a surprising fact, since social media can be very useful in several fields, e.g. volunteer recruitment, fundraising, media exposure and especially relationship management with the stakeholders of the organization. The literature analyses user engagement with contents published primarily on Facebook and Twitter, but the number of studies is low, since the term engagement itself is not entirely understood, which impedes the analysis (lines 58-65).*

*Previous research has also concentrated on large organizations, primarily based in the United States and, again, their organizational uses of Facebook and Twitter, which is especially popular in Anglo-American countries. Moreover, the researchers of these issues, when choosing a research sample, most often limited themselves to the method that can be described as "top-100-style sampling". It creates a research gap, as noted by Nah and Saxton denoting the underrepresentation of small- and medium-sized organizations in research samples. The use of random sampling is the solution to this problem, allowing more accurate inference via increased generalizability and simpler hypothesis testing. The appeal of both of these researchers only in a few cases was taken into account. Polish academic literature regarding the utilization of social media in the activities of non-profit organizations is also very modest. With a few exceptions, there has been practically no research conducted on the role of social media in building relationships with the community by non-profit organizations, especially by public benefit organizations (lines 65-78 ).*

*The analysis of academic literature also indicates that the vast majority of studies conducted so far have dealt with only a single social media channel. Therefore, there is a lack of research describing the coordination process of different communication channels. It seems that this issue is particularly important from a practical point of view, enhancing the stakeholder’s engagement (lines 79-83 ).*

*The present article aims to fill in at least part of that gap. Firstly, it contributes to the literature by examining the scope of the public benefit organizations’ use of the Facebook social network in managing relationships with the public (lines 84-86 ).*

*Secondly, the research described in the following article was part of a larger research project, which included an analysis of the use of Twitter in the activities of Polish public benefit organizations. The research methodology used for its analysis was then applied to Facebook research. The general conclusions formulated on this basis made it possible to identify differences in the organizational use of both communication channels and the development of practical guidelines for coordinating the utilization of different social media services in a non-profit organizations. Thirdly, random sampling was used to select the organization for the research, taking into account not only large organizations, but also medium and small organizations, meeting the appeal formulated by Nah and Saxton (lines 94-103 ).*

4. **Much more emphasis was placed on pointing out the relationship between literature review and hypotheses development. We added the following sentences to the literature review part of the article (some sentences were also moved or deleted).**

*Resource dependency theory is a starting point in explaining the reasons for the use of social media in the activities of a non-profit organization. As indicated by Mato-Santiso, Rey-García and Sanzo-Perez “this is because of non-profit organization’s endemic lack or shortage of resources relative to the size of their target groups’ needs (potential recipients, beneficiaries or users) and to the complexity of the social problems tackled” (lines 114-119).*

*The research that has investigated the use of social media for PR activities of non-profit organizations has so far been focused on only a few of its aspects. First, on the scope of social media usage and on which characteristics employed by the NGO increased the use of those services. Second, static elements of an organization’s profile on the social media site were analyzed. Third, the perception of the role of social media for an NGO by public relations practitioners were examined. Fourth, the contents published on social media sites and their impact on user engagement levels were analyzed. From the perspective of this article, the fourth aspect mentioned above is the most important which is associated with building public engagement (lines 147-155 ).*

*Engagement is defined as a dynamic multidimensional relational concept including psychological and behavioral features of involvement, connection, interaction, and participation designed in a way to enable the achievement of goals at an individual, organization or, social levels. Engagement can be understood as a process in which engagement is created, or cocreated, through communication or as a state or outcome that encompasses cognitive, affective, and behavioral dimensions. In the online environment, the theoretical foundations for the formation of engagement can be found in the work of, for example, Dhanesh, who also understands this concept as a cognitive, emotional and behavioral state in which the organization and the entities in its environment, sharing common interests in areas relevant to themselves, enter interactions which can be placed on a scale (lines 156–165).*

*The literature indicates that public engagement is influenced by a number of factors that may result from both the nature of the published content and the very characteristics of a non-profit organization. The first group of factors may include, for example, the function performed by the published content Research conducted by Lovejoy and Saxton, which made it possible to create a typology enabling the identification of the basic and specific function of content published in the online environment. This particularly concerned the Twitter content of American largest non-profit organizations. Other studies indicate the importance of communication tools used in single posts or tweets. It may be assumed that the more complex the post, the more likely the recipient would react (the presence of a video file, photos, links to external sites, hashtags or mentions in published content). Moreover, the length of the post or tweet and the time of its publication, e.g. a weekday or weekend, may also translate into a greater level of public engagement. The second group of factors concerning the features of the non-profit organization itself may also influence the public engagement of the content published on social media sites. These features may include the value of the organization’s assets or annual revenues, its age, type of activity, the number of followers of its social media profile (lines 182–197 ).*

*Kim and Yang treat pressing a “like” button as an affect-driven behavior, commenting on content as a cognitively triggered behavior and sharing as a combination of both. For example, the recipient of the content, by clicking on the "like" button, informs that he or she has a positive attitude towards the content and identifies with it to some extent. According to Saxton and Waters, liking a single post is the weakest form of engagement, as opposed to the number of comments, which should be prioritized from an organizational point of view, reflecting a significant commitment of the content’s recipient (lines 202–208 ).*

5. **In the subsection "Hypothesis development" reference is made to previous own research**

*This assumption also results from the research conducted earlier by the authors on the use of Twitter by Polish non-profit organizations (lines 250–251 ).*

*The importance of this factor is also indicated by the results of the Wallace and Rutherford studies, although the research sample in this case was only 160 non-profit organizations (lines 252–254 ).*

6. **In the subsection "Discussion" reference is made to previous own research. We added the following text**

*At first glance, the research results allowed framing similar conclusions as in the case of the previously conducted research on Twitter and its role in building relationships with stakeholders of Polish public benefit organizations. There are, however, some substantial differences. First, only in one case was Twitter employed as the major social media site rejecting all the rest. In the remaining cases, the organization, apart from Twitter, always used Facebook. If we additionally take into account the fact that the content on Twitter was characterized by a significantly lower reaction from the public (the number of likes, shares and comments was several or several times smaller than in the case of Facebook), its role in the case of Polish public benefit organizations is at best auxiliary. It even seems that due to numerous organizational limitations related to the shortage of qualified staff, lack of time and budget shortages, especially in the case of small organizations, it is impossible to effectively run more than one social media site. The only advantage of the organizational use of Twitter is the structure of posts in terms of their basic function. The content on Twitter was mainly informative, inherently inducing a minimal response from the public, because it does not serve this purpose. In the case of Facebook, the structure of analyzed posts in terms of the basic function was clearly different. The distribution of information, community building and call to action posts was very even. In this case, running two types of social media sites makes sense, however, due to a number of the above-mentioned barriers (human, financial and time), it is limited primarily to the largest entities. The research result proves that “free” technologies such as social media cannot be treated as an equalizer for smaller organizations in reaching an audience. Despite their seeming low entry barriers, effective social media use has more in common with other information technology investments. It means that the bigger the entity, the better. Thus, studies that show that smaller organizations tend to be more reactive and agile in adopting new information technologies cannot be confirmed (lines 723–748 ).*
